# The use of the comet assay for the evaluation of the genotoxicity of nanomaterials

**DOI:** 10.3389/fgene.2015.00239

**Published:** 2015-07-10

**Authors:** Amaya Azqueta, Maria Dusinska

**Affiliations:** ^1^Department of Pharmacology and Toxicology, Faculty of Pharmacy, University of NavarraPamplona, Spain; ^2^Health Effects Laboratory, Department of Environmental Chemistry, NILU-Norwegian Institute for Air ResearchKjeller, Norway

**Keywords:** comet assay, regulatory toxicity, genotoxicity, nanomaterials, OECD WPMN

## Introduction

The accelerating production and use of engineered nanomaterials (NMs) raises questions about the safety of this new technology. To avoid the possible hazards associated with NMs requires proper regulation. How should toxicity testing be addressed and can standard tests for assessment of safety of chemicals be applied to NMs? What are the major limitations of NM safety testing and is the current regulatory testing strategy suitable also for NMs? Can existing tests be fully adapted or should new methods be developed to suit the unique properties of NMs?

## Nanomaterials and nanoparticles

NMs are nanometer-scale materials that present at least one of their dimensions 100 nm or less. Nanoparticles are NMs with all three dimensions within 100 nm (http://ec.europa.eu/environment/chemicals/nanotech/index.htm#definition; Magdolenova et al., 2014). (In nanomedicine nanoscale particles larger than 100 nm are still considered as NMs.) The small size makes NMs very reactive, as the relative increase in surface area leaves a higher number of molecules to react with the environment. Thus, in their physical, chemical and biological properties, NMs are very different from the bulk material with the same chemical composition. On account of these unique characteristics they have found applications in a wide range of areas: technology, energy, construction, electronics, agriculture, optics, paint, textiles, food, cosmetics, medicine… The production of NMs has impressively increased in the last two decades and nowadays humans are exposed to an unknown amount of a great variety of NMs used in the production of daily life products.

## Genotoxicity assessment of nanonanomaterials

The same characteristics that make NMs interesting for many applications can also lead to toxicity. Thus concern about the potential harmful effect of NMs on human health has increased. NMs can enter the cell, interact with cell components and persist in cells with consequent chronic toxicity. A new research area that explores the potential toxicity of NMs in human and the environment is nanotoxicology. Within the nanotoxicology field, nanogenotoxicology studies the effect of NMs on DNA.

NMs can also enter into the nucleus, intentionally (i.e., in nanomedicine) or unintentionally, and there might interact with DNA, causing genetic damage (DNA breaks, altered bases or chromosomal damage). They can also reach the nucleus during mitosis and interfere with the microtubules, causing clastogenic effects. NMs can interact with cellular and mitochondrial membranes or alter mitochondrial function, provoking the production of reactive oxygen and nitrogen species and inducing DNA oxidation. Inflammation produced by NMs in tissues can also affect DNA. NMs can even induce genotoxic effects by depleting the antioxidant defenses or altering the DNA repair systems. All these events may result in pre-mutagenic lesions that can lead to mutations and possibly to cancer and other diseases.

Genotoxicity endpoints are crucial in assessing the safety of chemicals. The Organisation for Economic Co-operation and Development (OECD^1^) has published guidelines for several validated and standardized *in vitro* and *in vivo* methods including genotoxicity assays covering different endpoints (http://www.oecd.org/chemicalsafety/testing/oecdguidelinesforthetestingofchemicals.htm). It is clear that the strategies and the standardized protocols used for characterizing the potential toxicity of chemicals might not be fully suitable for assessing the safety of NMs. NMs can interfere with assay components or detection systems of standard toxicity tests (Guadagnini et al., 2015). In 2006, the OECD created a Working Party on Manufactured Nanomaterials (WPMN) with the aim to review the OECD guidelines for genotoxicity and assess their suitability for NMs.

In 2009 the OECD WPMN published a report recommending the bacterial reverse mutation (Ames test) (OECD TG 471^2^), mammalian chromosome aberration (OECD TG 473^3^) and mammalian cell gene mutation (OECD TG 476^4^) tests for *in vitro* testing; and the mammalian erythrocyte micronucleus (OECD TG 474^5^), mammalian bone marrow chromosome aberration (OECD TG 475^6^) and mammalian liver unscheduled DNA synthesis (UDS) (OECD TG 486^7^) tests for *in vivo* testing (OECD, 2009^8^). However, the Ames test is not suitable for NMs because there is limited or no penetration through the bacterial wall. Thus it is not surprising that NMs exhibiting a positive response in *in vitro* mammalian cell tests have shown negative results in the Ames test (Landsiedel et al., 2009; Doak et al., 2012; Jomini et al., 2012; Woodruff et al., 2012). In the case of the *in vitro* micronucleus test, the interaction between cytochalasin B and NMs is a limiting factor. Cytochalasin B inhibits cytokinesis and is used to generate the binucleated cells but it also inhibits endocytosis, an important mechanism of uptake of NMs into the cell (Doak et al., 2009; Gonzalez et al., 2011). NMs were also seen in the slides when high concentrations of NMs were tested (Pfaller et al., 2010) though this does not seem to be a problem.

Last year, the OECD WPMN published a new report about the genotoxicity evaluation of NMs (OECD, 2014^9^). The Ames test was not recommended for the investigations of the genotoxicity of NMs for the reason explained above. Modification of the *in vitro* micronucleus assay was discussed to ensure the exposure of the cells to the NMs in the absence of cytochalasin B.

Although a lot of effort is being made to develop a testing strategy to assess the genotoxicity of NMs in a reliable way, a consensus on regulatory requirements is still needed. According to the last OECD WPMN report there is a need for an assay that identifies and characterizes the DNA damage induced by secondary mechanisms (e.g., oxidative stress induced by inflammation) (OECD, 2014). Moreover, a complete strategy to assess the genotoxicity of NMs should cover different mechanisms and endpoints including assays to detect strand breaks and altered DNA bases.

## The comet assay in genotoxicity testing

The comet assay is widely used in *in vitro* and *in vivo* genotoxicity testing. It measures DNA strand breaks and alkali-labile sites in virtually any eukaryotic cell including cells isolated from tissues. Its modification with DNA repair enzymes, which convert the specific lesions to breaks, makes the assay more versatile (e.g., formamidopyrimidine DNA glycosylase, FPG, detects 8-oxoguanine and other purine oxidation products). The *in vivo* comet assay, in its standard version, has been validated and the OECD guideline was published last September (OECD TG 489^10^); this assay can be applied in many animal tissues, a great advantage when organ-specific toxicity is expected or investigated. The role of the *in vitro* comet assay in regulatory toxicity is currently not defined but efforts are being made to validate it. Nevertheless, it is recommended as an appropriate test under the Registration, Evaluation, Authorisation and Restriction of Chemicals Substances programme of the European Commission (REACH), is accepted by the European Food Safety Authority (EFSA) and is widely used for screening novel cosmetics and pharmaceuticals. It is also the most used assay in assessing the genotoxic potential of NMs. Magdolenova et al. (2014) reviewed genotoxicity techniques used in 112 papers, published from 2000 to 2012, where the potential genotoxicity of NMs was studied. Similarly, Azqueta et al. (2014) reviewed 102 papers where the genotoxicity of NMs with a potential application in medicine was assessed. According to the results of both reviews (Table 1), where the authors of this paper were directly involved, the comet assay and the micronucleus test are the most used techniques *in vitro* and *in vivo*, the comet assay being the most used in *in vitro* and the micronucleus test in *in vivo* studies.

**Table 1:** **Results obtained by Magdolenova et al. (2014) and Azqueta et al. (2014)**.

	**(Magdolenova et al., 2014) 112 papers**	**(Azqueta et al., 2014) 102 papers**
*In vitro* studies	94	81
**Comet assay**	**58**	**52**
**Micronuclei assay**	**31**	**30**
**Chromosome aberration test**	**10**	**9**
**Ames test**	**13**	**9**
**γ-H2AX by immunostaining**	**−**	**9**
*In vivo* studies	22	16
**Micronuclei assay**	**14**	**11**
**Comet assay**	**9**	**6**
Sporadic techniques	Chromosome aberration assay *in vivo*, gene mutation assay, sister chromatid exchange, γ-H2AX assay and others.	Chromosome aberration assay, gene mutation assay, sister chromatid exchange, γ-H2AX assay by immunostaining *in vivo* and others.

*Note that some papers can include results from both in vitro and in vivo studies and also different assays*.

Some interactions of NMs with the comet assay have been described though most of them are hypothetical. Some authors have described the presence of NMs in the comets, which implies that they were also present during the performance of the assay and could have interacted with the naked DNA inducing artificial additional damage (Stone et al., 2009; Karlsson, 2010). However, Magdolenova et al. (2012) found with 5 NMs that their presence in the gel does not affect the comet tail. Karlsson et al. (2015) discussed different possibilities of interference of NMs with the assay and concluded that under normal experimental conditions the additional damage is unlikely to be significant. NMs present in the comets could also interfere with the staining of the comets. Karlsson et al. (2015) suggest that, though there is no indication of this phenomenon, the visual scoring of the comets (rather than computerized image analysis) can be useful.

Interference of FPG with the comet assay (Kain et al., 2012) is also unlikely when applied correctly in the test (Magdolenova et al., 2012). On the other hand, caution is needed with photocatalytic NMs as they can induce additional breaks when the slides are exposed to normal light during their processing (Karlsson et al., 2015).

The comet assay has not been mentioned by the OECD WPMN as a potential appropriate test for testing NMs. The *in vitro* version of the assay does not have an OECD guideline yet though an *in vivo* versions was accepted in September 2014 (OECD TG 489), about 2 months before the publication of the OECD WPMN report on genotoxicity evaluation of MNs (OECD, 2014). The comet assay is considered as an indicator test detecting intermediate DNA lesions that can be repaired or fixed into mutations. Nevertheless, both *in vitro* and *in vivo* comet assays can complete the strategy to assess the genotoxicity of NMs since with the lesion-specific enzymes DNA lesions such as oxidized bases can be detected, additionally to DNA breaks. Moreover, the *in vivo* version is also suitable to detect DNA damage induced by secondary mechanisms such as oxidative stress induced via inflammation in several organs.

## Conclusion

Nanotechnology promises enormous benefits to society but also brings new challenges. One of them is the safety of new materials, and consequently there is a growing need for NM toxicity testing, Recent regulations based on hazard assessment of chemicals are not fully fit for purpose for testing NMs as current methods to assess NM toxicity do not always take into account the specific features of NMs. For example, some OECD-recommended tests for genotoxicity (Ames test), are not applicable, or need modification to avoid interference of tested NMs with the test system (micronucleus test). The comet assay has proved to be a sensitive and relatively simple method to study specific DNA lesions such as single and double strand breaks, oxidation and alkylation lesions or cross links. It is so far the most used method in nanogenotoxicology and has great potential to be included in a test battery due to its robustness, versatility and reliability.

### Conflict of interest statement

The authors declare that the research was conducted in the absence of any commercial or financial relationships that could be construed as a potential conflict of interest.

## Footnotes

^1^OECD guidelines for the testing of chemicals; http://www.oecd-ilibrary.org/environment/oecd-guidelines-for-the-testing-of-chemicals-section-4-health-effects_20745788

^2^OECD. Test Guideline 471. Bacterial reverse mutation test, 1997. In OECD Guidelines for Testing of Chemicals.

^3^OECD. Test Guideline 473. *In vitro* mammalian chromosome aberration test, 2014. In: OECD Guidelines for testing of chemicals.

^4^OECD. Test Guideline 476. *In vitro* mammalian cell gene mutation test, 1997. In: OECD Guidelines for testing of chemicals.

^5^OECD. Test Guideline 474. Mammalian erythrocyte micronucleus, 2014. In: OECD Guidelines for testing of chemicals.

^6^OECD. Test Guideline 475. Mammalian bone marrow chromosome aberration test, 2014. In: OECD Guidelines for testing of chemicals.

^7^OECD. Test Guideline 486. Unschedule DNA synthesis (USD) test with mammalian liver cells *in vivo*, 1997. In: OECD Guidelines for testing of chemicals.

^8^OECD. 2009. Preliminary Review of OECD Test Guidelines for their Applicability to Manufactured Nanomaterials. Series of Safety of Manufactured Nanomaterials No. 15.

^9^OECD. 2014. Genotoxicity of manufactured nanomaterials: report of the OECD expert meeting. Series of Safety of Manufactured Nanomaterials No. 43.

^10^OECD. Test Guideline 489. *In vivo* mammalian alkaline comet assay, 2014. In: OECD Guidelines for testing of chemicals.
